# Domestic Hygiene

**Published:** 1881-08

**Authors:** 


### POPULAR SCIENCE DEPARTMENT.

### PROFESSOR F. DONALDSON, M. D., ON DOMESTIC HYGIENE.

*Effects of Foul Air.*—We are frequently met with the reply, to any advice offered as to the danger of rebreathing air, that thousands of people live indoors and live for years. This is true. Yet it is not the less true that they are poisoned, because there is an adjustment of the organism to the medium by a gradual depression of the functions. The activity is reduced. Bernard's experiments showed that a vigorous, healthy bird would perish instantaneously in air which would sustain the enfeebled bird for an hour. As Lewes remarks with force: "There is a wonderful elasticity in the organism, enabling it to adapt itself to changing conditions; but a frequent depression of the functional activity must be injurious, and fatal if prolonged." If we take any small animal, a mouse for instance, and force it, after putting it into a close box, to breathe exclusively air exhaled from our lungs, it will soon faint, and if we persist in our cruelty, it will die. See the pale faces and ill looks of children who sleep with their heads under bed-clothes. Sir James Simpson (quoted by Kingsley) tells of a Christmas frolic where thirty-six persons danced all night in a small room, with low ceiling, keeping the doors and windows shut. The atmosphere of the room was noxious beyond description, and the effect was that seven of the party were soon seized with typhus fever, of which two died. At the Grotto del Cane, near Naples—where Mr. Bergh does not reside—a poor dog is kept to be stupefied, for the amusement of visitors, by the carbonic acid gas of the Grotto, and brought to life again by being dragged into the fresh air.

Our houses are not equal to the famous Black Hole of Calcutta, but it is only in a degree that they differ. We have shown how they are contaminated by injurious gases. It is the *organic matter* that we exhale, and the fermentive contagia which are produced to such an extent everywhere, which poison our house-atmospheres. Our house-sewerage is so defective, that insidiously we inhale poisons that propagate, even if they do not produce, typhoid fever, diphtheria, scarlet fever, &c.—filth diseases. *Sewers, it is undeniable, often become the channels through which contagious diseases are propagated.* The air from them, laden with specific, definite poison, easily penetrates, from its greater tension, into dwelling-houses, from imperfectly ventilated drains and imperfect traps. During the summer months, when the high temperature outside forces us to open our windows, these diseases are comparatively rare ; but as soon as the first autumnal cool weather comes we close our windows and put down our carpets ; then these diseases reappear and spread. It would appear that the germs, the ferments and organic dust lie dormant in the house-air during the Summer, and in the Fall spring into activity and spread by propagation in stagnant, foul air.

Some years ago we attended in consultation an adult with fatal diphtheria. One of the inmates of this large house left and stayed elsewhere for over a month, during which time the carpets had been taken up and some of the walls repapered, others whitewashed, and disinfectants had been extensively used. On her return to her old quarters, she went into a room in the back-building, distant some sixty feet from the room where the first patient had died. She took the disease and died in two days—a spark of contagion not removed carrying death before it !

We once went on duty, in the month of March, in a hospital where there had been a number of deaths from typhus fever; we ordered tents to be erected in the yard, and that each case, as it appeared, should be taken out of the building and laid in these tents. The weather was so cold and damp that large fires were kindled at the doors of the tents. Seventeen cases were so treated, and not one of them died. Why ? The medical treatment was very much the same as with the cases which had died indoors. It was the outdoor, pure, well-ventilated air which so wonderfully decreased the mortality.

The sick require more air than the well—at least from 3500 to 3800 cubic feet per head every hour. In the Dublin Lying-in Hospital, the deaths of new-born children amounted, in the course of four years to 2944 out of 7650 births. After a new and effective system of ventilation was adopted, the deaths in four years amounted to only 279. Thus, more than 2500 deaths, or one to every three births, must be attributed to bad ventilation.

In the badly ventilated prison of the Leopoldstadt, in Vienna, in the years 1834 and 1837, the proportion of deaths was 86 per 1000, out of which number 51.4 per thousand were due to consumption; while in the well-ventilated House of Correction, in the same city, the deaths were 14 per 1000 of which 7.9 were from consumption. It follows then that 43.5 cases per 1000 were deaths attributable to nothing but foul air.

Before the Crimean war the health of the English army was very bad; at least two soldiers died for every policemen. Foot Guards, 20.4 per 1000; Infantry, 17.8 per 1000; Metropolitan Police, 8.9 per 1000. The soldiers were picked men, better fed, clothed and lodged; but the air in the barrack dormitories was foul to a disgusting degree, and the air-space insufficient. Now, the death-rate amongst soldiers, thanks to sanitary improvements, is no more than that of the police, and not more than one-half it was before. So, in the French army, improved ventilation in barracks has given similar results. The British army, when in the Crimea, were lodged in tents during extremely rigorous weather, yet experienced a wonderful condition of health, such a thing as a cold being an unknown complaint; but when some of the men were placed in huts, which were much warmer, and in which there was a smaller circulation of fresh air, the sick rate rate increased, and coughs and colds began to appear. (Hartley.) Persons who during summer and winter sleep with their bedroom windows more or less open, cannot endure a night spent in a room with chimney closed and the window shut.

But there are poisons in the atmosphere which produce and promote disease, and yet are not known by any odors. "It is," says Dr. Simon, "of the utmost practical importance to recognize, in regard of filth, that agents which destroy its stink may yet leave all its main powers of disease-production undiminisned. It is certain that the ferments of diseaae, in doses in which they can fatally infect the human body, are infinitely out of reach of even the most cultivated sense of smell." The bacteria, the corpuscles, organic germs, and sporules, give us no warning; neither do the vibrios, micrococci, microzymes, nor the zooglœa, &c, Whether the germ theory of diseases of Dr Beale, or that held by Burden Sanderson, Lister or Tyndall, is correct or not, we know that the antiseptic method is wonderfully successful, and that the purer we keep the atmosphere the less virulent are the poisons. The poor suffer comparatively but little from enteric (typhoid) fevers. It is only the well-to-do who can have sewers in the houses, and sewers often become the real channels by which the contagion is propagated, in consequence of badly-trapped and imperfectly ventilated drains. During epidemics parents frequently keep their children in the house, for fear that by walking in the streets they may contract the diseases. Never was there a greater error. By staying indoors they breathe the same atmosphere, but with additional impurities—impurities which promote contagion.

We have not exaggerated in speaking of the impurities of house-air. That they may be removed there is no doubt; but that ordinarily they are not, even in houses of the better classes, is a clearly ascertained fact. The air in inhabited rooms, under the most favorable circumstances, can with difficulty be maintained in as pure a condition as the external air, yet the organic processes of the body are absolutely dependent upon the purity of the atmosphere. Fortunately for us, we do not remain in it always; but those who do, or who are much indoors, show its effects by their general weakness, their pallor, their bloodless condition, their susceptibility to atmospheric changes, their morning malaise, headaches, and dyspepsia. Indoor occupations predispose to all mal-nutritive diseases. How often it happens that some slight weakness operates as the entering wedge to the most serious diseases. Dr. Richardson's statistics showed that of 515 cases of consumption at his dispensary, more than two-thirds were of persons of indoor occupations.

Vitiated air is frequently the direct cause of consumption, the great scourge of the human race. Is it to be wondered at? When persons are deprived of sunlight, of exercise and of fresh air, their appetites fail, they lose their relish for food, and consequently they are insufficiently nourished. Disease necessarily follows.

*Deficient Sunlight.*—An influence of ill-health which is felt more especially in cities is the absence of sunlight. Many houses are situated due north and south, and the sun never reaches their front. The neighbors' residences or their own outbuildings keep off the rays from the other sides. Where the sunlight does attempt to intrude through the windows it meets with a persistent effort on the part of the mistress of the establishment to exclude it by means of inside curtains or outside shutters. The careful housewife wishes to protect the bright figures so beautifully woven in her carpets. It is no exaggeration to state that in many of the streets, not only of the older cities such as Rome and Paris, but of our own towns, the streets are so narrow and the houses so high that the sun never reaches either the sleeping or the sitting rooms. Again, a number of the modern houses of the rich are built three rooms deep in order to have the parlors and dining-room on one floor, and on each story above three sleeping rooms. A very convenient arrangement this for ease, but not for health. The middle room is necessarily a dark one. The parents consider this a nice arrangement for their children to be near them in case of sickness, but it never occurs to them that a room so far removed from the sun's rays is an unhealthy place in which to put them. They certainly would not put their flowers there unless they wanted to bleach them. It is not only from the residences that we have the beneficent orb's influence so sedulously excluded, but the school-houses are so situated that the children can scarcely even see the sun's rays, unless it is to teach them some fact of science in connection with it. In our places of business and warehouses we find the same disregard of the simplest hygienic knowledge of the value of sunlight. Many mercantile firms transact their business in narrow streets in the older parts of our cities where sunlight is never seen, unless probably for a short time at noon and then only in the middle of the street. It cannot get into the lower floors, or indeed anywhere where active business transactions are conducted. Even if the streets are wide the business floors are so many feet long that the sun can only enter a short distance. Ground space is so valuable in the thronged streets that every spot, where it is possible to put a house, is built upon, and every part of it is made to yield a revenue. We have in the business portions of our cities underground rooms, five, ten, and sometimes twenty feet below the surface, in which frequently not only goods are stored, but clerks are at work at their desks. In these, as far as the light of heaven is concerned, it is perpetual darkness.

There is light there, but not the light that nature intended they should have. The gas light enables the clerks to write and keep their accounts but at a fearful expense, not to the proprietors, but to the clerks themselves. The health of their bodies suffers more than if they were in darkness, for, in addition to the absence of the sun's healthful influence, they are compelled to breathe an atmosphere which is almost stagnant, rendered impure by their own breaths, and, what is still worse, by the very means used for illumination. How long can the air remain pure when there are several persons, each consuming sixty gallons an hour, and a number of gas-jets, each of which destroys air as much as eleven men—660 gallons per hour? But this is not all. In winter, when the windows must be closed to keep out the dampness of the surrounding ground, stoves are frequently used to keep the underground quarters comfortable. It is estimated that an ordinary stove will consume 15,000 gallons of air per hour. These basement rooms are considered very desirable for business purposes, and bring in high rents. They are much sought after for banking establishments, and singularly enough for life insurance companies, who do not hesitate, in insuring the lives of outsiders, to take the lives of their clerks and employes. Thus it is that not only in the principal cities of Europe, even in Italy, "where nature wears her brightest attire," hundreds of thousands of persons carry on their trades in almost utter darkness, but also in the cities of this progressing country of the new world. The sunlight is considered an enemy by the fair sex, who appear to be continually on their guard lest it should reach their faces. They avail themselves of all sorts of material to put over their pretty features to protect them from the sunlight. They consider the ruddy color of health unbecoming, and they think that a woman is more ladylike when her skin is transparent and bloodless. If their medical adviser undertakes to expostulate with them, he will find that he has undertaken a thankless office, and he may even be so treated as to discover that the sex is not always properly called the *gentle sex.*

If a leafy shoot of any plant be bent down without injury, so as to reverse the position of the faces of the leaves, the latter will twist upon their petioles and turn their upper surfaces to the light. Shoots of jasmine and ampelopsia when trained in a dark room will twist around to face the light. In Darwin's new work on "Power of movement in plants" we find the wonderful avidity with which the vegetable creation seizes upon light. It acts almost as if it had intelligence. *Thus the plants show a higher appreciation of sunlight than the better half of the highest in the scale of animals.*

What is this sunlight which is intentionally or thoughtlessly kept out of our houses? It is nothing more or less than the source of all force and consequently of all life, animal and vegetable. It was so made by the Creator, when he said, "Let there be light, and there was light." In the eloquent words of Sir David Brewster, "Light is the very life-blood of nature, without which everything in nature would fade and perish utterly." (Richardson.)

It is unnecessary to recite all the facts in nature that have established our belief that the various forces manifested in the actions of animal life trace their origin to that emitted by the sun, and that plants are the media for fixing the solar force for converting actual into latent or potential energy. Light influences the chemical changes made through food and air. Molecular rest is prevented in living beings by sunlight, and molecular activity constitutes life. Every one admits the necessity of a full exposure to the light of day to insure vigorous and healthy growth in almost all plants, the leaves and flowers of which follow the direction of the sun throughout its whole course from east to west. *Sunlight is as necessary for the color of animals as of plants. It is essential for the preservation of health and the attainment of the full development of the animal frame.* Without light, the mother coloring principle of the organism, the hæmoglobuline, is rendered pale, and the lessened intensity of the beautiful scarlet color is observable to all. The intensity of the red color of the red globules has been demonstrated to be owing to the quantity of oxygen they contain, and they have great avidity for oxygen and absorb it readily whenever they come in contact with it. The nutritive functions of the body depends upon this oxygen. Is it astonishing, then, that children and clerks shut out from the light, and workmen in tailor shops, millinery garrets, and mines should be pallid and ghastly, their muscles soft and flabby, and their nervous system relaxed and irritable?

We must bear in mind the effect of sunlight in dissipating dampness and preventing musty smells which are so promotive of disease. There is no surer way of developing a predisposition to consumption, or if their should be no hereditary tendency, of actually creating this disease. Constitutions thus trifled with have no power to resist the causes which bring about inflammation and mal-nutritive results. Our daily experience demonstrates the justice of this conclusion. In the country it is almost impossible to exclude the sunlight, and the occupations of those who live in the rural districts are not such as shut them out from the light. *Are we not right in considering absence of sunlight as a powerful influence in developing disease?*

Sunlight, my friends, has not only the beneficial effect of promoting health, but it destroys organic poisons, such as those of scarlet fever, diphtheria, measles and typhoid fever. "A physician in New Orleans, during the fearful epidemic of yellow fever, reported that there was six times as many cases of yellow fever on the shady as there were on the sunny sides of the street; a similar account was given of the prevalence of cholera in Buffalo, in 1849. Doctors Downes and Blount, in a paper read before the English Royal Society stated from actual experiments, that light prevents or retards the development of bacteria and of the microscopic fungi associated with putrefaction and decay. Dr. Richardson found that the virulent snake poison, woorari, was rendered inert when exposed to the sun's rays. Darkness is not only unhealthy upon our bodies, but it is injurious to the proper workings of our minds and our spirits. They are not lightened and refreshed as they should be. The mind is saddened in a home not flushed with light, and the mind being saddened, the whole physical powers soon suffer; the heart beats languidly, the blood flows slowly, the breathing is imperfect, the oxidation of the blood is reduced, and the conditions are laid for the development of many wearisome and unnecessary constitutional failures and sufferings.—(Hartshorn.)

Perhaps you have all heard the old Italian proverb: "Where the sunshine does not enter the doctor must."

Do our housewives recognize the beneficial influence of sunlight? If so, why do they so persistently shut it out from their halls, their parlors and their bed-rooms? It is no exaggeration to say that we stumble as we go through them. The outside shutters, the inside shutters, woolen curtains, inside blinds and lace curtains, all for one purpose to shut out an influence necessary to health. Even in regard to sick rooms, where infectious diseases are giving off germs to poison others, people will not listen to their medical advisers. Surely they are the slaves of the fashion of the hour. They would be offended were we to say "that they love darkness rather than light;" but they certainly utterly ignore habitual healthful influence of sunlight. "They put," says Richardson, "their flowers in their windows that they may see the light and healthy bloom. Are not our childrenworth many flowers? They are the choicest of flowers."

Then, my friends, open your shutters, put aside your curtains and let us have light. It will show us where dirt is that it may be removed. It will cheer us in our troubles of mind and body, and it will feed us with its influence, and leave no poisonous vapors behind it. It will promote health and strength, and cause to raise our hearts in thankfulness to Him who created it for our good.

